# Health Promotion and Wellness Initiatives Targeting Chronic Disease Prevention and Management for Adults with Intellectual and Developmental Disabilities: Recent Advancements in Type 2 Diabetes

**DOI:** 10.1007/s40474-018-0142-5

**Published:** 2018-06-23

**Authors:** L. Taggart, M. Truesdale, A. Dunkley, A. House, A. M. Russell

**Affiliations:** 1 Institute of Nursing & Health Research, Ulster University, Room 12J19, Shore Road, Newtownabbey, Co Antrim BT37 0QB Northern Ireland; 2 School of Health and Social Care, Edinburgh Napier University, Edinburgh, Scotland; 3 Diabetes Research Centre, University of Leicester, Leicester, England; 4 Leeds Institute of Health Sciences, School of Medicine, University of Leeds, Leeds, UK

**Keywords:** Intellectual and developmental disability, Type 2 diabetes, Prevention programs, Education self-management programs

## Abstract

**Purpose of Review:**

The aim of this paper was to review the recent international developments in health promotion and wellness initiatives targeting chronic disease prevention and management for adults with intellectual and developmental disabilities (IDD) targeting type 2 diabetes (T2D).

**Recent Findings:**

There has been one diabetes prevention program (STOP) and two self-management T2D education programs (DESMOND-ID; OK diabetes) adapted for this population. All three programs have been adapted from other theoretically informed and tested programs developed for the general population. Each program has employed co-design and co-production techniques with all stakeholders. The three programs all target the high-risk lifestyle factors that can lead to T2D and contribute to poor glycaemia control, and have undertaken randomized-feasibility studies, the results of which are promising.

**Summary:**

This paper shows that any health promotion and wellness initiatives need to be tailored and reasonable adjustments made in order to address this population’s cognitive impairments and communication difficulties.

## Introduction

‘Wellness’ is an important concept for all populations in society including those with an intellectual and developmental disability (IDD). Wellness initiatives can prevent the onset of many chronic health diseases, and can also assist individuals to cope or manage when they have developed a chronic health condition. That said, it can also be challenging to promote wellness in light of also having a chronic health condition. This paper reviews the most common chronic medical condition in adults generally, as well as in those with an IDD, type 2 diabetes (T2D), from a wellness standpoint. We were interested in exploring what is known about efforts to promote wellness to prevent diabetes onset as well as efforts to promote wellness for individuals with IDD who have a T2D diagnosis.

## Type 2 Diabetes in the General Population

T2D is a universal chronic health condition, and figures are predicted to increase as the world’s population continue to age [1]. It is estimated that 1.5 million deaths annually are associated with T2D globally. People with T2D can experience a range of secondary health conditions including heart disease, strokes and renal disease. The costs for managing such health conditions are extensive and growing for many countries [2], who are being challenged to both prevent the spread of diabetes and to promote the self-management of T2D. Dabelea et al. (2014) [3] reported that there has been a 30% increase in the number of adolescents who have been diagnosed with T2D between 2000 and 2009, thereby indicating that T2D is no longer a condition associated with ageing.

## Management of Type 2 Diabetes in the General Population

Being diagnosed with a chronic health condition such as T2D, it is therefore important to ensure that you maintain both good physical, and psychological, wellbeing in order to prevent developing complications, leading to other chronic health conditions. Promoting wellness and health promotion initiatives to prevent T2D developing is based upon targeting unhealthy lifestyle risk factors. Similarly, managing T2D is also based upon targeting healthy lifestyle modifications (i.e. dietary change aimed especially at weight loss, increased physical activity, smoking cessation, blood pressure and lipid management, foot care, monitoring of blood sugars and medication adherence) [4, 5]. However, many non-disabled patients find this management strategy difficult to implement and sustain. Both wellness and health promotion initiatives, and also T2D management programs, vary in delivery style and structure, and specific areas being targeted. Gillies et al. (2007) [6] suggested that the risk of developing T2D can be reduced by 60% if we tackle the leading lifestyle risk factors that contribute to the aetiology of this health condition.

Supporting people to have better control of their diabetes has been shown to decrease their risk of developing complications, which in turn can diminish the financial and human costs of diabetes [4, 7]. For the many people who have developed T2D, there is strong research evidence to show that diabetes self-management programs, based upon a theoretical framework, can reduce the risk of the complications of T2D [4]. The need for structured diabetes education programs for T2D has been recognized internationally and has been given high priority on many government healthcare agendas [1, 4, 7].

## Diabetes and People with Intellectual and Developmental Disabilities

People with IDD are also living longer and it is projected that this longevity will continue [8]. As a consequence, people with IDD may be more susceptible to developing age-related chronic health conditions such as T2D. Two systematic reviews [9, 10] reported that prevalence figures for T2D in this population were estimated to be 2–3 times more likely compared to their non-disabled peers as a result of the following:

- *Genetic*: specific genetic/chromosomal conditions (e.g. Down syndrome) are more likely to be correlated with higher obesity levels
- *Lifestyle*: people with IDD are more likely to have poorer diets, be less physically active, lead more sedentary lifestyles and have higher levels of obesity
- *Health related*: higher levels of multi-comorbidity, high levels of anti-psychotic medication prescription, greater dependence on family/paid carers, lack of uptake of health screenings/surveillance and a lack of reasonable adjustments being made by healthcare and specialist diabetes services [11–13].

MacRae et al. (2015) [9] found that within their systematic review, the average age of being diagnosed with T2D was 40 years; this is younger compared to those without disabilities (51 years). This highlights the greater need for not only health promotion and wellness initiatives but also self-management diabetes education programs. Lennox et al. (2007) [14] in Australia and Taggart et al. (2013) [11] in Northern Ireland both found a considerable number of people with an IDD living in the community with T2D, with high levels of obesity, which was neither identified nor effectively managed by their primary healthcare team. Bryant et al. (2017) [15] in England also found high levels of obesity and physical inactivity in those adults with IDD and T2D, but encouragingly found that many of their respondents wanted help in changing their diet and managing their diabetes better.

## People with IDD, Diabetes Health Promotion and Management Initiatives

There have been few theoretically informed and robustly evaluated interventions that focus on health promotion and well initiatives to prevent people with IDD at high risk from developing T2D [11, 14, 16–19]. Similarly, there have been few theoretically underpinned and robustly evaluated self-management education programs for those adults with IDD and T2D [11, 12, 14]. It can be challenging for those with IDD and T2D to access, and be involved in, existing national self-management education programs. Furthermore, both prevention and self-management programs in the general population do not consider how to involve family or paid caregivers in such groups [11, 17, 19–21]. These general self-management programs have neither recognized nor addressed the specific challenges posed by this population’s cognitive deficits, communication difficulties, low levels of literacy skills and learning styles, as well as higher lifestyle and cardiovascular risk factors [12]. Wilson & Goodman (2011) [22] in England found that adults with mild/moderate IDD and co-morbid physical health conditions (i.e. diabetes, arthritis, hypertension, etc.) could successfully participate in chronic disease self-management programs if such programs were modified.

## Aim

The aim of this paper was to review the recent wellness initiatives in diabetes prevention and T2D management for adults with IDD. The paper found one diabetes prevention program (STOP) and two recently published self-management T2D education programs (DESMOND-ID; OK diabetes) adapted for adults with an IDD. We reviewed the theoretical underpinnings of each program, the program development and content, and the current state of the evidence concerning these programs.

## STOP Diabetes Prevention Program

### Development of the STOP Diabetes Prevention Program

The ‘STOP Diabetes’ program was developed as a multi-component lifestyle behaviour change initiative for the prevention of T2D targeting cardiovascular risk factors in adults with IDD [23–25]. A multi-disciplinary team were brought together comprising expertise in the field of IDD and in mainstream diabetes. Program development utilized an iterative approach, informed by current guidance on developing complex interventions by the Medical Research Council (MRC) [26] and intervention mapping [27].

The first phase of development combined (1) stakeholder interviews, conducted with service users/carers and health professionals; and (2) reviews of key IDD-specific research, existing interventions and behaviour change literature. The second phase of development involved two pilot cycles of testing, evaluation and refinement of the curriculum. Modifications made to the curriculum content, delivery and resources were based on observations made during sessions and qualitative feedback. During this pilot phase, a quality development process was also developed for assessing intervention fidelity [23]. The third and final curriculum derived was subsequently tested in a small feasibility study [25].

### Theoretical Models Underpinning

The theoretical underpinning of the STOP program [24] was informed by the frameworks reported by Bazzano et al. (Healthy Lifestyle Change Program) [28], and elements of social cognition models such as the Theory of Planned Behaviour and Reasoned Action [29, 30]. Key aspects incorporated into the program included using concrete kinaesthetic and observational methods of learning; preparatory work with individuals (prior to attendance and at the start of each session); reflection on personal levels of risk; self-monitoring (i.e. through pedometers and/or food diaries); exploring barriers and solutions to making lifestyle changes; and flexibility in the delivery and resources to account for different levels of IDD. Self-efficacy was identified as a key aspect of behaviour change; however, it is recognized that people may not have complete independence over their activities, such as buying and cooking their own food.

The behavioural goals and lifestyle messages included in the STOP program were based on two existing prevention programs [31–33] that were developed for the general adult population. However, the emphasis in the STOP curriculum is on more generalized behaviour goals linked to nutrition and physical activity, rather than prescriptive targets. These included increasing physical activity and/or reducing sedentary behaviour (sitting time); choosing smaller portions; reducing consumption of sugary drinks and foods; reducing consumption of processed foods and ready meals; choosing healthier snacks/treats; and increasing fruit and vegetable intake.

### The STOP Diabetes Program

The STOP diabetes education program was aimed at adults with mild/moderate IDD who were at high-risk of developing T2D and/or cardiovascular disease (based on impaired glucose regulation or BMI of ≥ 25 kg/m^2^). Participants needed to be willing/able to attend group education sessions and able to walk for at least short distances (with or without walking aid). The program consists of one initial carer session, followed by seven joint education sessions for the person with IDD and their carer/spouse/partner (2.5 h each, held weekly). The sessions were designed to be delivered in a community setting by two educators (i.e. a registered ID nurse and a diabetes specialist with an educational background) and one support person. The initial carer session provided an overview of the program, allowed the carer to meet the educators, explored their role in supporting the service user and ask any questions they may have. The curriculum for the participant and carer sessions together explored different topic areas (broadly related to health, physical activity and nutrition) each week, and builds on/consolidates learning from earlier sessions (see Table 1).

**Table 1:** STOP diabetes education program

Week	Key elements of each session	Resources/activities	Theories
Week 1			
Topic area 1 What is health? Being healthy and unhealthy	Explore what concept of being healthy means to individual Explore behaviours linked to health Develop images that represent healthy and unhealthy characters to be used throughout the program	• Poster—healthy and unhealthy characters • Images to prompt recognition and recall • Summary cards for main messages	SRT TPB
Topic area 2 What can go wrong with my health?	Explore health consequences of behavioural and lifestyle choices Explore behavioural and lifestyle choices that promote health Have an opportunity to express emotional responses to choices the characters make	· Images to prompt recognition and recall	SCT SRT TPB
Week 2			
Topic area 1 This is me and health checks my doctor or nurse will do	Create an image that represents the individual and their behavioural and lifestyle choices Be aware of health checks a doctor or nurse may do Be provided with their own results/risk factors Be aware of which results may be a problem to own health Place stickers on health profile to plot individual results	• Personal activity sheet • Images to prompt recognition and recall • Personal health profile with photograph • Biomedical data • Coloured stickers	SCT SRT TPB
Topic area 2 What can I do to stay healthy?	Explore impact of results/risk factors on own health Express any emotions/concerns relating to their results Recall consequences of behavioural and lifestyle choices Explore behavioural and lifestyle choices relating to own risk factors Choose and record lifestyle changes on personal poster Record level of confidence to make changes	• Images to prompt recognition and recall • Confidence activity sheet	SCT SRT TPB
Week 3			
Topic area 1 Being active	Explore what being active means Be aware of health consequences of being inactive Explore benefits to health of moving more and sitting less Experience using a pedometer to measure steps	• Images to prompt recognition and recall • Physical activity record • Pedometer • Walking activity	SCT SRT TPB
Topic area 2 Me and my activity	Experience a short walking activity and record steps in activity diary Identify ways to increase activity/step count and/or reduce sitting time Record level of confidence to carry out chosen goal Create reminder or prompt for chosen goal to promote engagement Record activity in a diary	• Images to prompt recognition and recall • Confidence activity sheet • Create prompt cards, fridge magnet, or send a postcard	II SCT SRT TPB
Week 4			
Topic area 1 How did I do with my activity?	Reflect on current level of activity Reflect on feelings related to level of activity Explore own and other group members barriers to physical activity Explore strategies for overcoming barriers Highlight the increase in steps from short periods of activity Identify new steps or activity goal for coming week	• Interactive dice game to explore barriers • Physical activity record • Walking activity (optional)	SCT SRT TPB
Topic area 2 Changes I can make to be healthy	Recall behavioural and lifestyle changes that influence risk factors Be aware of the impact of unhealthy behavioural and lifestyle choices over many years—facilitated by using story book Recall personal behavioural/lifestyle choices recorded in session 2 Reflect on progress with choices Explore sources of support for recording food, drinks and snacks over the next week outside session	• Personal lifestyle and behaviours activity sheet • Story book • Food diary	II SCT SRT TPB
Week 5			
Topic area 1 How did I do with my activity? Eating well, eating healthy	Reflect on activity levels over last week Generate ideas for overcoming barriers Plan new activity/step goal Recall main health messages Identify foods linked to a healthy lifestyle Identify foods related to being unhealthy Be aware of possible impact of high fat, sugar and large portions on health Be aware of possible benefits of healthier food choices and smaller portions	• Physical activity record • Walking activity (optional) • Food models and images to support recognition and recall • Food sort task • Stickers	II SCT SRT TPB
Topic area 2 Changes I can make to eat well and eat healthy	Recall food messages from earlier session Record personal confidence to make change to food choices Identify 1 or 2 small changes to make based on personal food diary Create personal prompts to behaviour change	• Food models and images • Food diary • Create prompt cards, fridge magnet, or send a postcard	II SCT SRT TPB
Week 6			
Topic area 1 Where am I with my activity?	Reflect on activity levels over last week Generate ideas for overcoming barriers Plan new activity/step goal Recall the main food messages and possible impact of food choices	• Physical activity record • Walking activity (optional) • Food diary • Food bingo game	II SCT SRT TPB
Topic area 2 How am I doing with my eating well, eating healthy?	Reflect on the food diary Identify successes and barriers to making changes to food choices Explore own and other group members’ barriers to making changes Explore strategies for overcoming barriers and how to reward personal success Identify sources of support and plan a new food goal	• Food diary • Barriers board game • Amend or create prompt cards, fridge magnet, or send a postcard	II SCT SRT TPB
Week 7			
Topic area 1 What have I learnt?	Reflect on activity levels and food diary Review overall program and raise any outstanding concerns or questions Recall main learning points and revisit associated activities Identify successes and barriers to making changes	• Food diary • Activity diary • Healthy/unhealthy character posters • Personal lifestyle and behaviours activity sheet • Images to prompt recognition and recall	II SCT SRT TPB
Topic area 2 What can help me to keep going with changes to my food and activity levels?	Record changes on personal activity worksheet Explore possible solutions to barriers and strategies to help support maintenance of changes Set new goals and individual strategies to help Record personal confidence to carry out chosen goals Explore sources of support to help achieve goals Celebrate success	• Worksheet to record changes made • Confidence activity sheet • Postcards (written to self-to send in few months), fridge magnets, flashcards and stickers • Prompt card to give to carers for help • Course attendance certificates	RP SCT SRT TPB

II, implementation intentions; RP, relapse prevention; SCT, social cognition mode; SRT, self-regulation theory; TPB, theory of planned behaviour

### Evaluation of the STOP Program

Dunkley et al. (2017) [25] undertook a feasibility study of the STOP program which involved five adults with IDD and T2D, and their family/paid carers. Both the adults with IDD and their carers indicated that it was possible to engage them in the education program and collect pre-and post-outcome measures. Measuring physical activity and sedentary behaviour was less successful, as some service users declined to wear the wrist-worn accelerometers. The study was not designed/powered to detect differences in outcome measures between baseline and follow-up; Dunkley et al. (2017) [25] suggested a general trend towards improvement in biomedical measures.

Feedback and observations collected during the pilot phases suggested that the program was acceptable to both the adults with IDD and their family/paid carers [23, 24, 32]. The adults with IDD reported that they enjoyed the sessions and it helped them to make and sustain changes to their diet and physical activity levels. Most of the adults with IDD were supported by carers; their input was valued by the educators, and the family/paid carers reported that the program had helped them to encourage and enable the service users to make behaviour changes. Although dietary changes and increase in physical activity were reported to be made by the participants, caution must be taken as this study was only based on the feedback of five adults with IDD. This initiative has not been further tested.

## DESMOND-ID Type 2 Diabetes Self-Management Education Program

### Development of the DESMOND-ID Program

DESMOND-ID is an adaptation of the original DESMOND (Diabetes and Self-Management for Ongoing and Newly Diagnosed) structured self-management T2D education program [33, 35]. The original DESMOND program (aims/objectives, content, structure, curriculum, length of sessions, resources, health action plans and interactive sessions) were adapted by Taggart and colleagues, involving adults with IDD and T2D [36]. The team consisted of experts in IDD and mainstream diabetes, who explored the original DESMOND program with a small group of adults with IDD and T2D; adaptations were made to the program. This new adapted education program mirrored the main aims and objectives of the original DESMOND program.

The adapted DESMOND-ID education initiative was a 7-week program designed to be delivered in two parts. The first part (session 1) was for family/paid carers and spouses/partners only, and delivered 1-week in advance of the commencement of the main program. This 2-h session focused on what is T2D, introducing the aims of the DESMOND-ID program and role of carers in supporting the person with an IDD over the forthcoming 6 weeks. The second part of the education program (sessions 2–7) was delivered over 6 weeks, two and a half hours per session, 1 week apart and is for both the person with ID and their carer/spouse/partner together. Table 2 below provides an outline of the diabetes educational curriculum. The DESMOND-ID program is delivered by two educators (i.e. health care professionals, nurses, dietitians). The educators received 2 days DESMOND core training, which covered a range of topics including patient philosophy, theories of learning and supporting behaviour change, as well as one additional day of training in the DESMOND-ID curriculum.

**Table 2:** DESMOND-ID diabetes educational curriculum

	Session
Day 1	
Welcome and introduction	25 min
My story with diabetes (part 1)	15 min
My body and diabetes	20 min
Break	15 min
What is diabetes	35 min
What did I learn today and preparing for next week?	10 min
Day 2	
Welcome back	20 min
My story with diabetes (part 2)	15 min
What diabetes does to your body?	25 min
Break	15 min
Food and blood sugar	35 min
What did I learn today?	10 min
Day 3	
Welcome back	20 min
Knowing what your blood sugar levels mean	35 min
Break	15 min
Being active	40 min
What did I learn today?	10 min
Day 4	
Welcome back	20 min
Heart and circulation problems: what can I do to keep healthy (part 1)	40 min
Break	15 min
Other diabetes health problems: what can I do to keep healthy (part 2)	35 min
What did I learn today?	10 min
Day 5	
Welcome back	20 min
Food and fats	35 min
Break	15 min
Making healthier food choices	40 min
What did I learn today?	10 min
Day 6	
Welcome back	20 min
Diabetes health action plan: what will I work on?	35 min
Break	15 min
Keeping my plan going	35 min
Important questions and celebration of achievement	15 min

### Theoretical Underpinnings of DESMOND-ID

The program is based on a series of psychological theories of learning and education: Leventhal’s Common Sense Theory (i.e. illness representation, illness beliefs), Dual Process Theory (process of learning) and Social Learning Theory (i.e. self-efficacy). The philosophy of the program was founded on patient empowerment, as evidenced in published work [33, 34]. Development of the program followed a systematic approach, guided by the current MRC framework for developing and evaluating complex interventions [26].

### Evaluation of the DESMOND-ID Program

Taggart et al. (2017) [37] undertook a UK national randomized-feasibility controlled trial of the DESMOND-ID diabetes education program (*N* = 19) versus standard routine care (*N* = 20) in Northern Ireland, Scotland and Wales. All the participants had T2D, mean age was 54.7 years. Most participants (69%) were supported to attend the DESMOND-ID program with a family/paid carer. Over 90% of adults with an IDD attended between 4 and 6 sessions and 94% of carers attended between 6 and 7 sessions. Biomedical data (HbA1c, BMI, blood pressure, perceptions and severity of diabetes) were collected at baseline and 3 months follow-up.

This study found that with reasonable adjustments, it was possible to identify, recruit and obtain consent from adults with a mild to moderate ID, and deliver the DESMOND-ID education program. Taggart and colleagues found that for those participants in the DESMOND-ID group, their HbA1c reduced statistically from 66 mmol/mol to 57 mmol/mol (*p* < 0.05), compared to an increase for those participants in the control group (61 mmol/mol to 65 mmol/mol).

Through focus groups post-intervention [37], the six DESMOND-ID educators reported the program addressed the lack of and sometimes incorrect understanding of T2D and its implications among both the participants with IDD and their carers. More importantly, the program explained how to better self-manage their T2D through a healthy diet, increasing physical activity and medication adherence. The educators also reported that the DESMOND-ID diabetes education program was developed at the appropriate level for those with mild/moderate ID. However, the program was not suitable for those with a severe/profound IDD.

## OK Diabetes Self-Management Education Program

### Developing the OK Diabetes Program

‘OK Diabetes’ was a supported 1–1 self-management education program. Supported self-management for chronic health conditions such as T2D is now well established internationally [38]: what is needed is not therefore a ground-up development of a newly theorized treatment but modification of existing approaches. The OK diabetes program took a broadly problem-focused approach, seeking to identify specific barriers to good self-management and to help the individual marshal personal and social resources (especially instrumental social support) to overcome those barriers. Against this largely social and interpersonal background, individual change techniques such as goal setting could be modified to suit the participants’ needs [39, 40].

Using problem structuring and priority setting, preliminary versions of the supported self-management package, including not just format and content but tailoring (for easy reading, visibility for those with poor acuity and so on), were discussed initially by the research team. Finally, we considered guidance on reasonable adjustments to healthcare designed to ensure access for people with an IDD. At each stage, there were regular consultation meetings with service users, carers and their representatives.

### OK Diabetes Program

The program had four standardized components with associated materials, delivered by diabetes specialist nurses. How they were delivered depended on participant and supporter characteristics and preferences (see Table 3). A training program was delivered over three sessions of face-to-face contact with the nurses. An additional session on mental capacity assessment was also included since the nurses had no prior disability experience. In each case, the whole intervention was delivered over a maximum of four visits and the nurses met after each visit. Based on this experience, early versions of the intervention were modified in format to make them easier for use by the nurses. The nurse worked through the elements of supported self-management diabetes education with the participant with IDD, explaining how to use materials and suggesting initial actions and activities. Further contact was negotiated with the person with IDD and T2D. Overall, a total of three to four meetings of 30 to 60 min, over 6 to 8 weeks, were be provided, followed by telephone support and advice.

**Table 3:** OK diabetes program

1. *Establishing the participant’s daily routines and lifestyle*: This included current diet and activity routines, participation in daytime social activities or work, shopping and food preparation, current self-reported health and self-management.	
2. *Identifying all supporters and helpers and their roles*: A key supporter and other helpers were identified where possible. Key supporters and other helpers were given written information about the project and if they agreed to support a goal set by the participant they were given a written reminder of their role.	
3. *Setting realistic goals for change*: The main aim was to avoid prescribing change in the way of good dietary practice or other lifestyle change, but to support goals suggested by the person with diabetes that were specific, simple and achievable given the person’s current routines and social support, and consonant with their willingness to make change.	
4. *Monitoring progress against agreed upon goals*: We devised a simple system that did not depend on high levels of functional literacy, using tear-off calendar sheets on which participants noted goal attainment in a yes/no format.	

### Evaluation of the OK Diabetes Program

House et al. (2018) [40] undertook an individually randomized-feasibility controlled trial of the OK diabetes program vs usual care in England, randomizing 41 adults with mild-moderate IDD to the OK diabetes program and 41 adults to routine care [41]. Self-management sessions lasted on average 45 min and largely took place in the participant’s home (92%). The most frequent goals identified were to increase physical activity and to make dietary changes.

Records of program adherence were kept [42]. Of the participants randomized to the OK diabetes group, 83% attended all sessions required to cover all the components of the intervention at least once: which meant from two to four sessions, with over three quarters of all participants (78%) attending at least three sessions. A summary of engagement was reported by the nurse who delivered the intervention: 58% participants were deemed to be very engaged with the sessions and 30% with the materials; 37% were reported to have a very engaged supporter (consenting or non-consenting); and 44% had a further or different person (other supporter, partner or family member) who was engaged in the intervention implementation.

Although the primary aim of the study was not to assess efficacy, clinically important outcomes, as part of determining the feasibility of a definitive trial, were collated. Six-month outcomes were obtained from 94% of participants. In the OK diabetes program, 35% of those who received supported self-management either lost > 5% body weight or dropped HbA1c > 5.5 mmol/mol. House et al. (2018) [40] results suggest that the OK diabetes program is practical and acceptable, and recruitment and retention rates propose that a definitive trial is possible. Qualitative feedback suggested that important elements included face-to-face contact with the nurse, practical problem-solving involving supporters and goal setting. The authors concluded that despite four sessions being delivered, this was possibly too short, and future iterations should aim for more contact as well as a greater emphasis on weight reduction.

## Discussion

There have been strong criticisms that many health promotion and wellness initiatives targeting any chronic disease prevention and management targeted at people with IDD have little, if any, theoretical underpinnings and are not robustly evaluated [12]. This paper has provided a succinct review of three promising theoretically informed, diabetes prevention (STOP) and self-management diabetes education programs (DESMOND-ID, OK diabetes) for adults with IDD. Each program has (1) adapted their programs from mainstream prevention and self-management diabetes education programs; (2) utilized different individual change theories; (3) co-designed, co-developed and co-produced a program alongside adults with IDD and T2D, their carers and other stakeholders; and (4) undertaken a randomized-feasibility study in line with the MRC guidelines for evaluating complex interventions [26].

The STOP program focused on the prevention of diabetes; this program used a group education format over a 7-week period. The DESMOND-ID and OK diabetes programs both focused on the self-management of this chronic health condition; the former initiative was based on a group format over a 7-week period whereas the Diabetes OK program was based upon a 1–1 format over four sessions. All three programs used health professionals as the educators, and all three programs involved family/paid carers/spouses/partners. The results of all three programs are very promising in terms of their adaptation processes, program content, delivery and in their evaluations. However, The STOP program had only a sample size of five adults with an IDD whereas the DESMOND-ID and OK diabetes programs had 39 and 82 adults with IDD prospectively: further research is still required regarding their clinical and cost-effectiveness.

As the aetiology of T2D can be multi-causal (i.e. genetic, lifestyle risk factors, health access), any health promotion and wellness initiatives, and chronic disease management initiatives, must be tailored to the specific needs of the target population to improve a number of target outcomes (i.e. HbA1c, diet, weight, physical activity, smoking cessation, medication adherence, knowledge and attitudes, etc.). However, for those adults with IDD who have a cognitive impairment and communication difficulties, the development of such prevention and self-management initiatives is much more complex. Research also clearly illustrates that many adults with IDD are dependent upon family and paid carers to support them to make healthier lifestyle choices on their behalf [16–19]. All three programs have incorporated and welcomed family/paid carers and spouses/partners as part of the learning process to support the adults with IDD needed in making the appropriate healthier lifestyle choices.

Diabetes self-management education programs can be observed as complex regimes of self-care practices (restrictive, behaviourally challenging and complicated) in order to achieve optimal glycaemic control, and reduce potential acute and long-term complications. It can be questioned whether individuals with IDD given their cognitive impairments and communication challenges can adhere to such regimes. The studies by Taggart et al. (2017) [37] and House et al. (2018) [40] offer two distinct approaches to the self-management of T2D, using a group approach or a 1–1 approach. Both approaches were found to be encouraging; however, House et al. (2018) [40] acknowledged that the four 1–1 sessions needed to be extended. The STOP diabetes prevention program further illustrated that with reasonable adjustments and engaging with family/paid carers, people with IDD can adhere to such 7-week education regimes [43].

All three programs are limited to people with mild to moderate IDD, and who have a family/paid carer or spouse/partner who can provide support for them, although those with a more severe/profound IDD would not necessarily be able to engage: but their carers could still be involved to learn how to manage the person’s diabetes. Despite the favourable accomplishments of these three innovative health promotion, wellness and management initiatives targeting diabetes [25, 37, 40], there are many methodological and practical challenges that threaten research studies in hard to reach and recruit populations such as those with IDD. For example, identifying cases can be a challenge, since most adults with a mild IDD are generally not recorded on community registers and may not be registered on primary healthcare registers [12, 44]: this may be similar in many other countries.

## Conclusion

This paper provides a review of three promising theoretically informed health promotion and wellness and self-management initiatives. The three programs have successfully adapted and made reasonable adjustments/amendments for the cognitive and communication needs of this population that can be translated into other chronic disease conditions such as arthritis, asthma, cancer and coronary heart disease.

Recognizing and understanding the specific cognitive impairments of this population (i.e. memory, organizational skills, information processing, problem-solving, decision making, attention, understanding and orientation, self-efficacy, etc.) and communication difficulties is essential in adapting/modifying or developing any new health promotion and wellness initiatives. Lessons learnt from these programs include breaking the aims and objectives of such programs into understandable and achievable chunks; using concrete kinaesthetic and observational methods of learning; modifying the curriculum content, delivery and resources using easy read and pictures/symbols; self-monitoring (i.e. through pedometers and/or food diaries); and exploring barriers and solutions to making lifestyle changes. All three programs also understood the role that family/paid carers play in supporting the person with ID to interact in the health action plans of these programs between each session. The good management of T2D can lead to better glycaemia control and thereby diminish potential complications from occurring and preventing premature deaths [45].

Greater emphasis should be placed upon wellness and health promotion initiatives that can prevent people with IDD developing chronic health conditions such as diabetes, coronary health disease and cancer. Efforts should be placed upon wellness initiatives that promote healthy diets, increase physical activity and reduce sedentary behaviours, thereby targeting one of the significant health hazardous risk factors—obesity [1, 12]. This will include the reduction of over-medication. Likewise, importance should also be placed upon living with and managing a chronic health condition, wellness initiative that can further prevent additional health complications. We need further research into how people with IDD live well with a chronic health condition (i.e. how they cope, the impact upon their mental health), and how can services/systems meet this population’s needs.
